# Supplementary material for: MetaRibo-Seq measures translation in microbiomes
Source: Nat Commun. 2020 Jun 29;11:3268. doi: 10.1038/s41467-020-17081-z (PMC7324362; doi:10.1038/s41467-020-17081-z)
Supplement: Supplementary file 10 — Supplementary Data 7 [file 41467_2020_17081_MOESM10_ESM.zip › File2/Confidence_High_Taxonomy/31238_out.krona.html]

Javascript must be enabled to view this page.

members
magnitude
magnitudeUnassigned
count
unassigned
taxon
rank

31238\_out

4

superkingdom
2759

SRS075398\_contig\_number\_207
1


SRS012902\_contig\_number\_contig-100\_4076.4076SRS147139\_contig\_number\_contig-100\_3536.3536
2

1
superkingdom
2

phylum
1239
1

class
186801
1

1
186802
order

family
31979
1

genus
1485
1

1

SRS023829\_contig\_number\_1018
species
1720194
